# On Supposed Unsuccessful Cases of Vaccination

**Published:** 1802-08-01

**Authors:** 

**Affiliations:** Newington Butts


### *On supposed unsuccessful Cases of Vaccination; communicated by* Mr. Pears, *of Newington Butts.*

A Mis-ftatement refpecting a cafe of *Cow Pock* which has lately happened in St. George's Fields, having produced fome effect injurious to the Vaccine Inoculation, and alfo influenced the minds of fome parents fo far as to make them averfe to that *advantageous* mode, I take the liberty of tranfmitting the particulars, as they cannot be too generally known; and as relating to a cafe, wherein the Small-pock was actually fuppofed to have occurred *after* Cow-pock.

It would certainly be fufficient to remove all doubt refpecting the nature of the firft inoculation (for Cow-pock) to fay that it was performed by *Mr. Ring*, during his laborious and indefatigable exertions in propagating the advantages of Vaccine Inoculation; but as the circumftance alluded to was fo far diftinct tinct from that as to have been produced by a fortuitous occurrence one year and a half afterwards, it cannot be improper to recite the particulars, *as stated by the mother.*

*Mary Salt*, aged nine years and a half, was inoculated in both arms by Mr. Ring, about eighteen months fince. The puftule arofe, and proceeded in the ufual way, and others were inoculated therefrom by Mr. R.

On Tuefday, June 29th, this child "fickened for the Small-pock," as was fuppofed by the mother, from her having the ufual fymptoms, in confequence of her having caught the infection from another child, —— Jenkins, who had been kept *in the house* while labouring under that dreadful difeafe, and was only removed on the preceding day. (Monday, June 28.) The child was returned from fchool, much indifpofed, *and with an inflammation affecting both arms, in the places inoculated, and extending from the shoulder to the elbow.* An eruption afterwards appeared on the face, neck, arms, hands and body, exactly refembling the Small-pock. The opinion of Mr. Heaumes, a practitioner in the neighbourhood, was requefted, who thought it was *variolous*, as did alfo Mr. J. K. of the Borough, *several* other gentlemen, and Mr. Ring, who being informed of the circumftance by the mother of the child, on the following day, came to fee it in the evening, and (as the mother ftates) "thought it had a variolous appearance." Thefe gentlemen vifited the child every day. The puftules arofe inflamed, contained a watery fluid*, continued three days, (Monday, Tuefday, and Wednefday), and on the fourth, (Thurfday) difappeared, *when* all the Practitioners agreed that it was not Small-pock: a rafh alfo accompanied this variolous-like appearance.

At the fame time, another child, named *Mary Hull*, aged three years, who lived at the next door, had been inoculated with Mary Salt, by Mr. R. and who had frequently played with —— Jenkins during the Small-pock, had the fame kind of eruption as the above-named child, Mary Hull, with the fame appearances, continuance, &c.

The mother of *Mary Salt*, from whom I received the account, is "*not only perfectly satisfied that it was* NOT *the Small-pock which appeared upon her child, but is also much strengthened in her assurance of the* SECURITY *afforded by Cow-pock*, from fuch an expofure as her child was fubject to, with the one who laboured under the Small-pock." Both the children continue in good health.

It

* Mr. Heaumes has obligingly informed me that there was *not* any fluid, and that the variolous appearance was fo far gone on the fecond day as to induce a change in his opinion. The accompanying rafh had a morbillous appearance.

It is therefore a matter of ſome conſequence that the caſe ſhould be fairly ſtated, and the particulars fully known, ſince it *appears* to need accurate inveſtigation for refutation.

Inſtances of this kind, it is fortunate, are now occuring but ſeldom; and when they do occur, they are immediately aſcertained and *refuted.*

The *Cow-pock* is now very generally received and regarded, as an elegant writer has lately expreſſed it, "*as a friendly guest that tarrieth but a day unattended by danger; it departs and leaves behind it no ground for regret, but on the contrary much for satisfaction and joy at its visit, imparting to every individual who has received it into his constitution the blessing of stable security from one of the deadliest foes of the human species.*" See Booker's Sermon on the Cow Pock*, a mode of recommendation for which the Doctor is entitled to the THANKS of the medical profeſſion as a moſt effective way to its becoming that "*parochial* concern," which the author (at p. 11) ſo judiciouſly recommends and enforces, as tending ſo effectually to promote the reception of what he aptly thinks entitled to the *distinguishing* name of Kind Pock.

Attempts of this kind, by the regular and parochial clergy, would go far to produce that generally good effect which is ſo deſirable. The plain and earneſt addreſs of a zealous and ſound divine, herein imitating the example of that worthy dioceſan *Bishop Squires*, in his Sermon (on Inoculation) before the Small-pock Hoſpital, would effectually remove the prejudices and ſatisfy the minds of perſons in every ſtation of life †; and it is to be preſumed that the Medical Profeſſion would not be backward in acknowledging the obligation conferred, and the aſſiſtance rendered them by the regular Clergy on this as well as other undertakings of a uſeful and charitable nature.

*July* 19, 1802.

---

* *A discourse (addressed chiefly to parents) on the duty and advantages of* INOCULATING *children with* THE COW POCK, by Luke Booker, LL.D. Printed for Hatchard, 196, Piccadilly, 1802. 1s. 6d.; a Sermon which is entitled to the peruſal and acknowledgments of every regular practitioner, who could not fail to be pleaſed both with the ſolidity of the argumentation and the elegancy of its compoſition. The notes alſo contain much uſeful and important information.

† This has been done by ſome of the *country* Clergy.

